# “Off–On” fluorescent sensing of organophosphate pesticides using a carbon dot–Au(iii) complex†

**DOI:** 10.1039/c7ra13404e

**Published:** 2018-03-23

**Authors:** Miao Wang, Minmin Li, Jia Lu, Bei Fan, Yan He, Yatao Huang, Fengzhong Wang

**Affiliations:** Key Laboratory of Quality & Safety Risk Assessment on Agro-Products Processing (Beijing), Ministry of Agriculture Beijing 100193 China fanbei517@163.com wangfengzhong@sina.com; Institute of Food Science and Technology, Chinese Academy of Agricultural Sciences Beijing 100193 China; Tibet Academy of Agricultural and Animal Husbandry Sciences Lhasa 850000 China

## Abstract

Herein, we report novel “off–on” fluorescent sensing of organophosphate pesticides using a carbon dot (CD)–Au(iii) complex/acetylcholinesterase (AChE) system. The above sensor utilizes the quenching of CD fluorescence by Au(iii) and its subsequent recovery by thiocholine, which is generated by AChE-catalyzed hydrolysis of acetylthiocholine (ATCh) and effectively scavenges Au(iii). In the presence of organophosphates, the catalytic activity of AChE is inhibited, allowing these species to be quantified based on the decreased recovery of CD fluorescence intensity. The developed sensor was used to analyze a real pesticide-spiked sample (4.48 μM), achieving a recovery of 99.85% and exhibiting a linear response range of 0.45–44.80 μM.

## Abstract 2

Herein, we report novel “off–on” fluorescent sensing of organophosphate pesticides using a carbon dot (CD)–Au(iii) complex/acetylcholinesterase (AChE) system.

## Graphical abstract

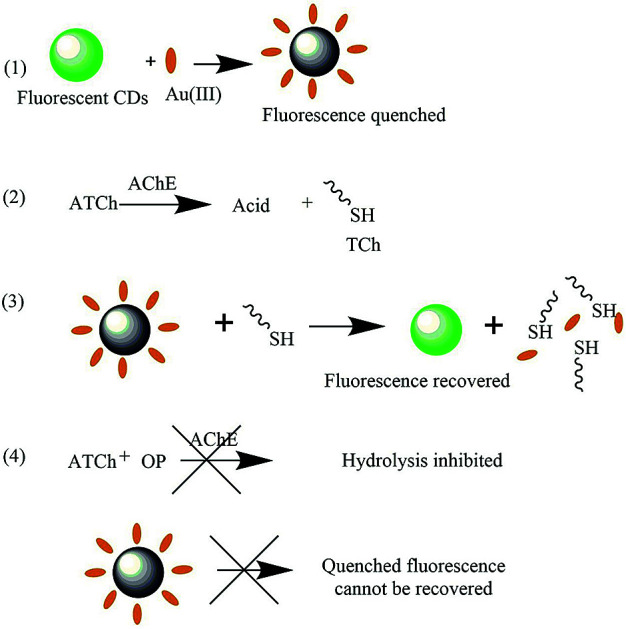

## 1 Introduction

Organophosphate pesticides find extensive applications worldwide, exhibiting acute toxicity upon ingestion and thus posing a serious risk to human health.^1,2^ The above pesticides mainly target acetylcholinesterase (AChE),^3^ inhibiting its catalytic activity and thus hindering the hydrolysis of acetylcholine (ACh, a neurotransmitter), with the accumulation of excess ACh leading to lethal overexcitation.^4,5^ However, the above activity inhibition can be successfully utilized in organophosphate pesticide sensing.

Carbon dots (CDs) have recently found numerous applications due to exhibiting excellent fluorescence, good biocompatibility, and low toxicity and cost.^6,7^ Recently, much effort has been directed at utilizing CD fluorescence or electrochemiluminescence to fabricate “off–on” or “on–off” sensors,^8–10^ which exhibit the advantages of fast response, easy operation, and clear signal recognition.^11^ Notably, metal ions such as Cu^2+^, Fe^3+^, Hg^2+^, K^+^, and Ag^+^ (ref. 11–15) are able to quench the fluorescence of CDs,^16^ allowing the sensing of heavy metals, organic pollutants in water, sugar in blood, and biomarkers in living cells.^17–20^

Herein, we report a novel “off–on” fluorescent biosensor for organophosphate pesticides, in which the CD fluorescence initially quenched by Au(iii)^20^ is recovered by thiocholine (TCh, an effective Au(iii) scavenger) generated by AChE-catalyzed hydrolysis of acetylthiocholine (ATCh) that is structurally similar to ACh. In the presence of organophosphates, the catalytic activity of AChE is inhibited, allowing these species to be quantified based on the decreased recovery of CD fluorescence intensity.

## 2 Materials and methods

### 2.1 Materials and reagents

Eggs were purchased from the Chaoshifa supermarket. AChE and ACh were purchased from Sigma-Aldrich (USA). H_2_SO_4_, NaOH, and HAuCl_4_ were sourced from Sinopharm Co. (China), and all other chemicals were obtained from the Beijing Chemical Plant. Deionized (DI) water was produced using the Milli-Q system (USA). Incubators and magnetic stirrer plates were purchased from IKA Instrument Co. (Germany). Fluorescence spectra were recorded on a HITACHI 2000 spectrometer (Hitachi Co., Japan), and Fluorescence Spectrometer FS5 from Edinburgh Instruments (U.K.). Transmission electron microscopy (TEM) imaging was performed using a JEM-2100 instrument (JEOL Co., Japan). The Fourier transform infrared spectrometer was Tensor 27 from Buruker. Co. *etc.*(Germany). X-ray Photoelectron Spectroscopy (XPS) was tested by using PHI Quantera SXM, from ULVAC-PHI (Japan).

### 2.2 CD synthesis

A procedure previously reported elsewhere^23^ was optimized and used to prepare CDs.

Fresh egg white (4 mL) was dissolved in DI water (40 mL) upon 3 min stirring, and a 10 mL aliquot of the obtained solution was transferred into a 50 mL flask, treated with 98 wt% H_2_SO_4_ (4 mL, slow addition!), agitated, and placed in a water bath (50 °C) for 2 h. Subsequently, the mixture was neutralized with 5 M aqueous NaOH and subjected to 5 min centrifugation at 5000 rpm. The supernatant was transferred into an Eppendorf tube and treated with dichloromethane (3 mL), with another 5 min centrifugation at 5000 rpm resulting in the extraction of the synthesized fluorescent carbon dots into the organic phase. The dichloromethane phase was separated and blown dry with nitrogen, and the thus obtained CDs were dispersed in DI water for utilization in the next step.

### 2.3 Characterization of CDs and CD/Au(iii) systems

The emission spectrum of carbon dots was scanned with different excitation wavelength (slit width = 10 nm). And the morphology of carbon dots was characterized by transmission electron microscope (TEM). The surface groups and elemental speciations were tested by FTIR and XPS respectively.

A series of Eppendorf tubes was charged with the abovementioned aqueous CD dispersion (20 μL), which was followed by the addition of 0.2 M phosphate-buffered saline (PBS, pH 7.0, 100 μL) and solutions containing different concentrations of Au(iii) ions. The resulting mixtures were diluted with DI water to 2 mL, incubated at room temperature for 8 min, and probed by fluorescence spectroscopy.

### 2.4 Organophosphate pesticide sensing

AChE-catalyzed hydrolysis of ATCh afforded TCh that could bind Au(iii) ions and thus recover CD fluorescence. Since organophosphate pesticides inhibit AChE, their presence resulted in slower TCh generation and thus decreased CD fluorescence recovery. A series of organophosphate pesticide solutions of different concentrations was added to the CD/Au(iii)/(ATCh + AChE) system to construct a calibration curve.

## 3 Results and discussion

### 3.1 Principle of “off–on” fluorescent sensing

AChE catalyzes the hydrolysis of *S*-acetylthiocholine iodide to TCh,^22^ which can effectively capture Au(iii) and thus recover the CD fluorescence quenched by the same.^20^

**Scheme 1:**
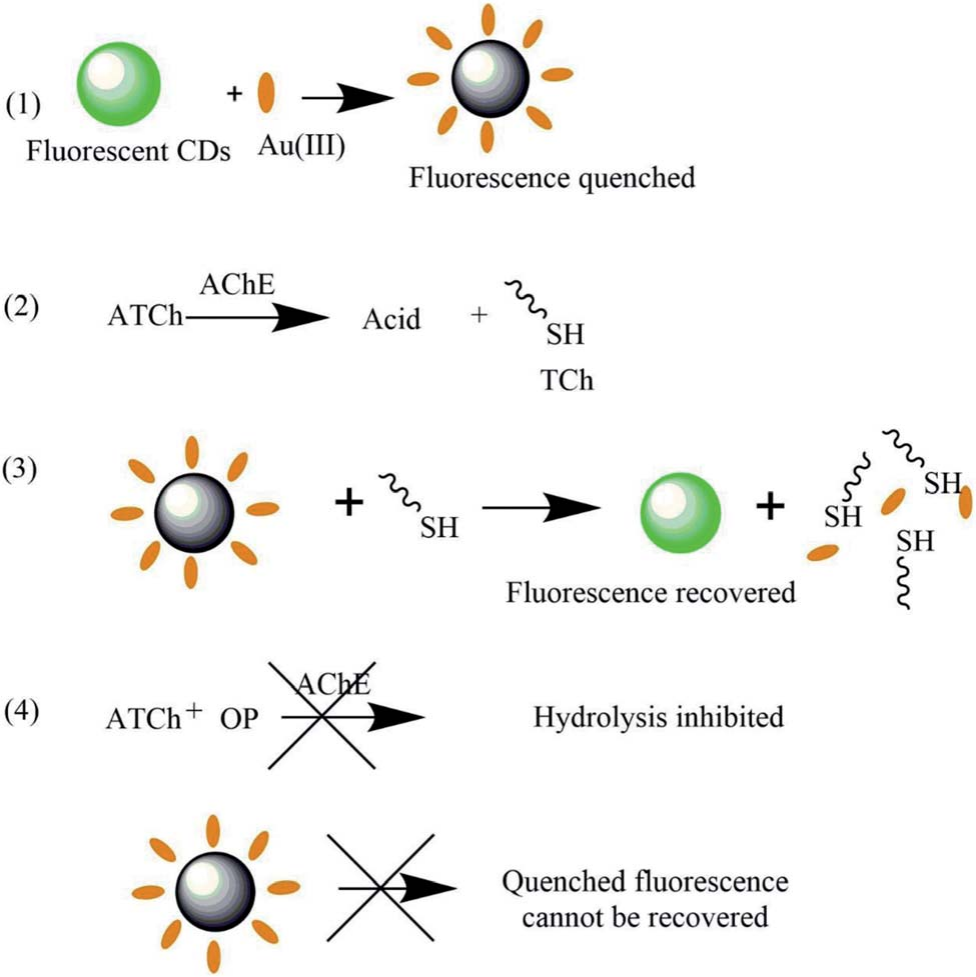
Mechanism of organophosphate pesticide sensing by the developed CD/Au(iii) sensor.

As mentioned above, organophosphate pesticides inhibit AChE, thus decreasing the rate of TCh generation and hindering fluorescence recovery, which was used for their detection (Scheme 1).

### 3.2 CD synthesis and properties

**Fig. 1:**
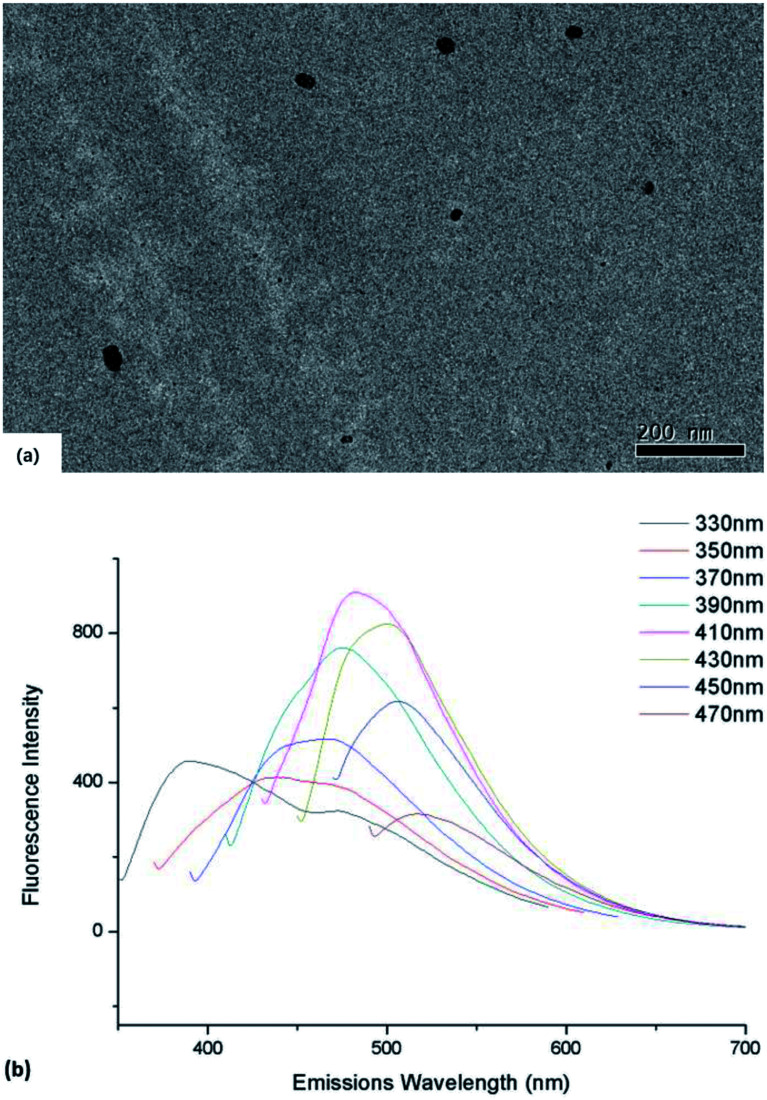
(a) TEM image of as-synthesized CDs dispersed in DI water (b) emission spectra of CDs varies with different excitation wavelength.

The synthesized CDs exhibited nearly spherical shapes (diameter ≈ 20–40 nm) and were well dispersible in DI water (Fig. 1a). With the different excitation wavelength, the emission wavelengths of bare CDs vary from 480 to 530 nm, respectively (Fig. 1b). According to the FITR spectrum (S.1†), the broad absorption bands near 3400 cm^−1^ was the character peak of *ν*(O–H) and *ν*(N–H). The peak at 1720 cm^−1^ should be *ν*(C=O). And the peak at 1600 cm^−1^ should be scissor bending vibration of H_2_O. The peaks of 1395 cm^−1^ and 1150 cm^−1^ refer to the bending vibration of *δ*(CH_2_) and *ν*(C–O–H). Composition of carbon dots synthesized was analyses by using XPS. While insensitive to H, the result (S.2†) showed that the C, O, N and S were at present of 62.84, 12.53, 22.56 and 2.07%, respectively. The fluorescence quantum yield (QY) of carbon dot was 7.06%, tested by Fluorescence Spectrometer FS5 (Edinburgh Instruments, U.K.). However, with excitation light with different wavelength, the emission light wavelength also varies. Regarding the steady of testing and the harm of UV light to AChE, we choose 470 nm as the excitation wavelength in following experiments.

### 3.3 Fluorescence quenching by different metal ions

**Fig. 2:**
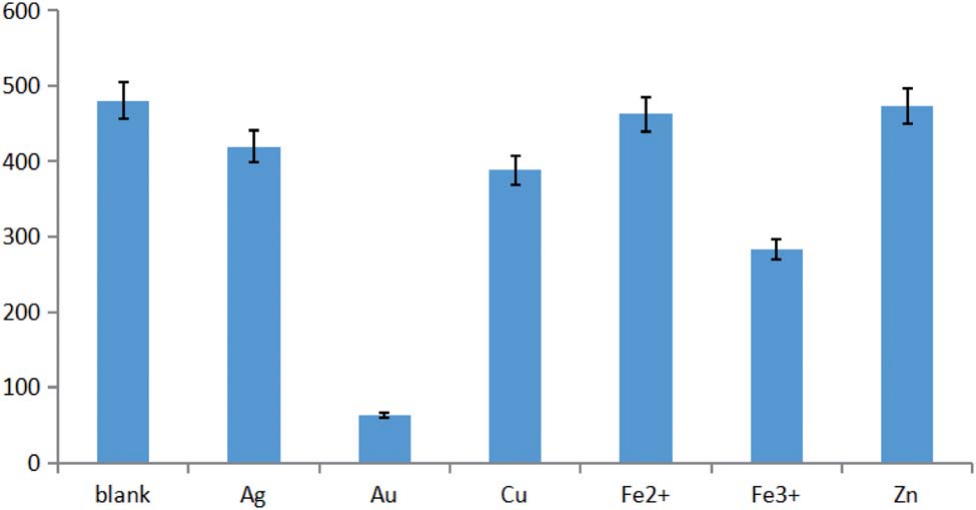
Fluorescence quenching effect of different ions.

Metal ions exhibit a well-documented ability to quench the fluorescence of CDs. The results obtained for selected ions (Fig. 2) revealed that the most significant quenching was observed for Au(iii), with increasing Au(iii) concentration thus causing a decrease of CD fluorescence.

**Fig. 3:**
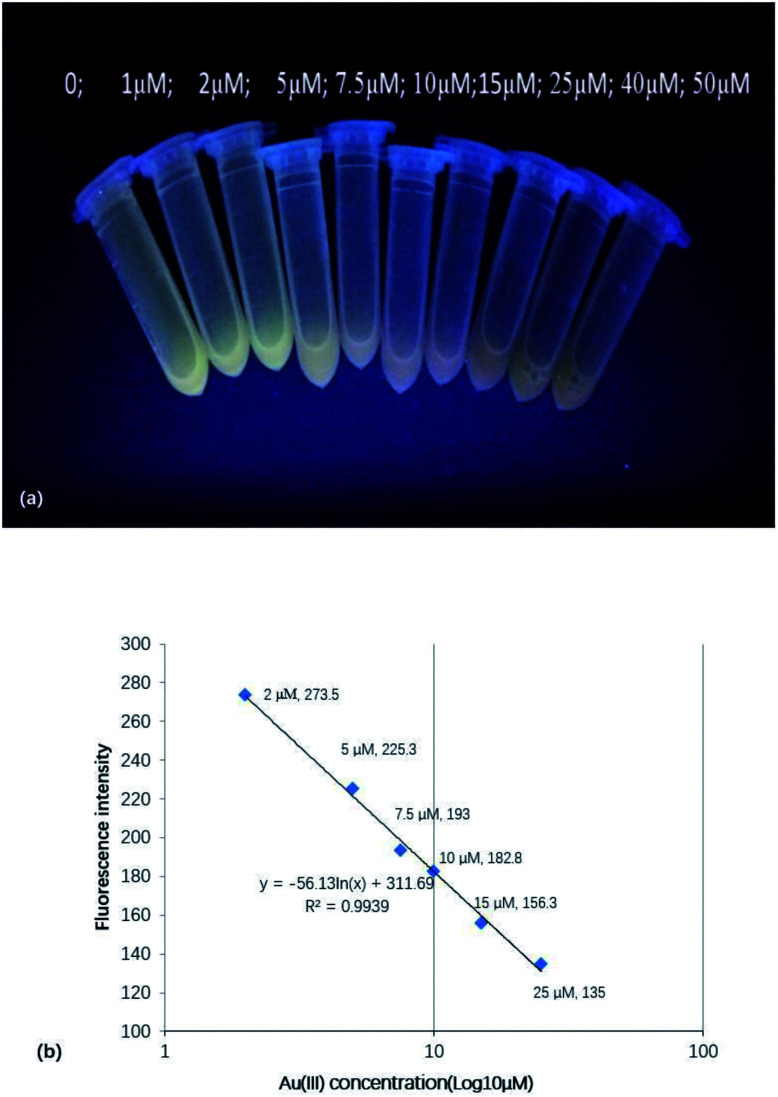
(a) Visually observed changes of CD fluorescence (excitation at 365 nm) upon the addition of Au(iii). (b) Dependence of fluorescence intensity on Au(iii) concentration.

When excitation was performed at 365 nm, the fluorescence changes could be observed by the naked eye, as shown in Fig. 3a. Fig. 3b shows that Au(iii) could effectively quench CD fluorescence, with the fluorescence intensity at the maximum emission wavelength linearly decreasing with increasing Au(iii) concentration.

### 3.4 Fluorescence recovery in the presence of ATCh and selected anions

As mentioned above, AChE can catalyze the hydrolysis of *S*-acetylthiocholine iodide into TCh^23^ that can effectively capture Au(iii) and thus recover CD fluorescence.^21^

**Fig. 4:**
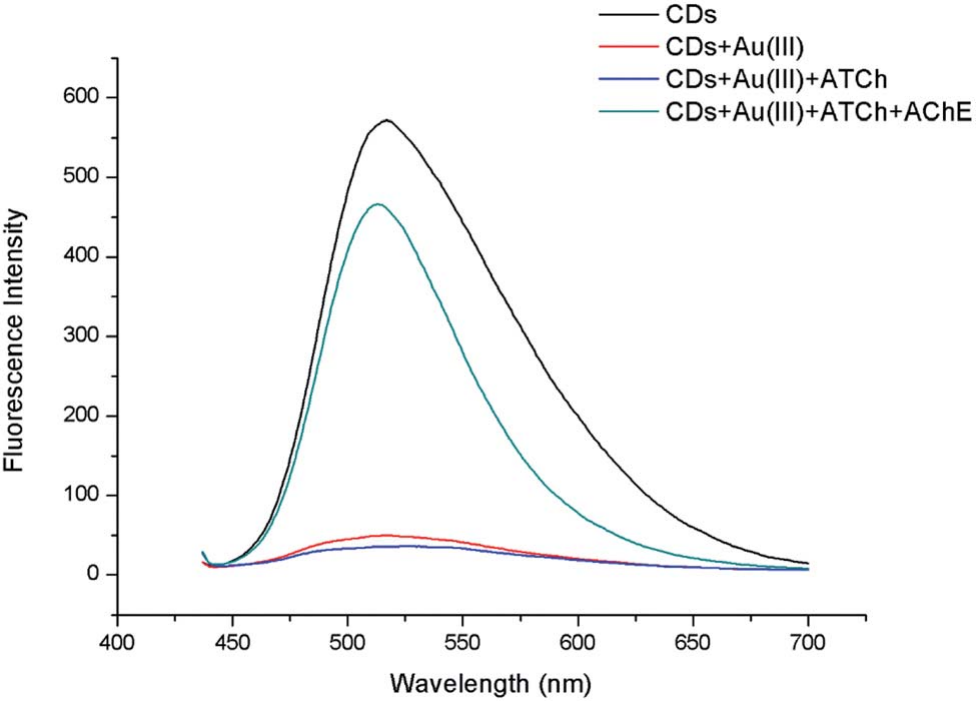
Fluorescence changes of CD-containing systems observed upon the addition of Au(iii).

The amount of produced TCh, and thus, the extent of CD fluorescence recovery, was expected to be proportional to the quantity of added ATCh. A preliminary experiment was carried out to prove feasibility of the mechanism. The naked CDs, CDs quenched by Au(iii), CDs with Au(iii) and ATCh, CDs with Au(iii) and ATCh hydrolyzed by AChE were compared by using fluorescent spectrum scanning. As Fig. 4 shown, the fluorescence of CDs can be significantly quenched by adding 50 μM Au(iii). However, the CDs Au(iii) ATCh hydrolyzed by AChE got stronger fluorescence as the TCh caught most of the Au(iii). And it makes the fluorescence of system just a little bit lower than naked CDs. Therefore, this experiment proved the assembled scheme was correct.

**Fig. 5:**
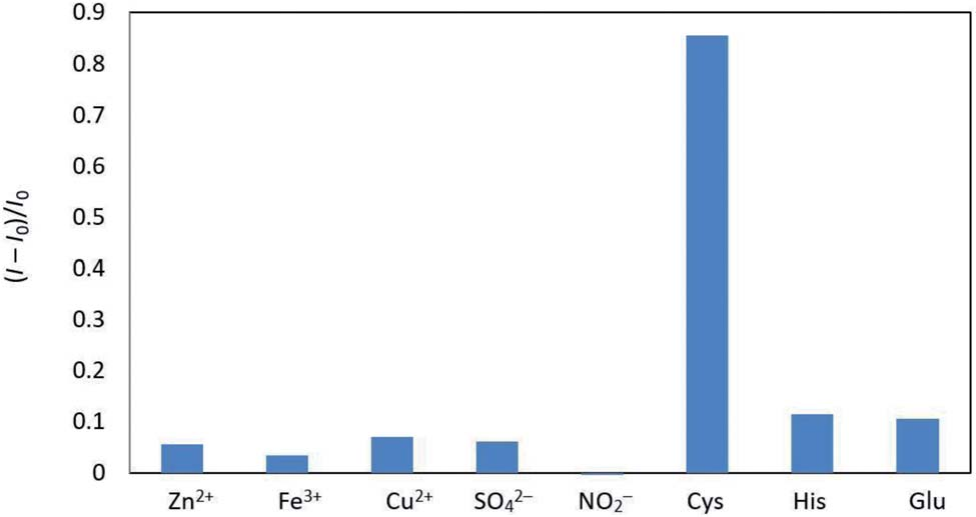
Influence of amino acids and common anions on fluorescence quenching in the CD/Au(iii) system.

Next, we investigated the influence of potential interferants on fluorescence quenching in the CD/Au(iii) system, showing that most inorganic ions and amino acids exhibited no apparent effects (Fig. 5). However, the addition of cysteine significantly increased the fluorescence of the above system due to this compound possessing an SH group capable of scavenging Au(iii), as outlined in Scheme 1.

### 3.5 Optimization of ATCh concentration

**Fig. 6:**
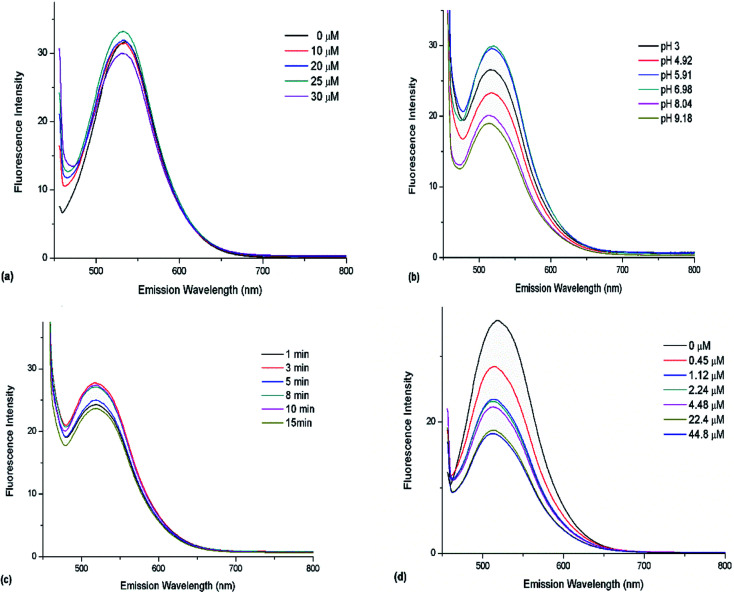
Optimization of enzymatic hydrolysis parameters for better sensing. Effects of (a) ATCh concentration, (b) pH, and (c) hydrolysis time on sensor fluorescence recovery. (d) Inhibition of sensor fluorescence recovery by different concentrations of organophosphate pesticides.

With the increasing of TCh coming from hydrolyzed ATCh, more and more Au(iii) were captured. However, this linear dependence was only observed in a certain range. It can be observed in Fig. 6a that the certain range was 0–25 μM. Thus, the optimal ATCh concentration in the reaction system was determined as 25 μM.

### 3.6 Effects of pH

Optimal concentrations of Au(iii) and CDs were selected to investigate the effect of pH, which was adjusted by PBS. Subsequently, the products of AChE-catalyzed ATCh (1 mL, 50 μM) hydrolysis performed at 37 °C for 8 min were added to the CD/Au(iii) system, and fluorescence spectra were recorded after 8 min (Fig. 6b). As a result, optimum performance was observed at pH 6.98, which is similar to the pH of maximal AChE activity.^24^

### 3.7 Effects of hydrolysis time

The effect of hydrolysis time was investigated at pH 6.98. As described above, the products of AChE-catalyzed ATCh (1 mL, 50 μM) hydrolysis performed at 37 °C for 1, 3, 5, 8, 10, and 15 min were added to the CD/Au(iii) system, and fluorescence spectra were recorded after 8 min (Fig. 6c). The optimal hydrolysis time was determined as 8 min and was used in subsequent experiments.

### 3.8 Determination of organophosphate pesticides

**Fig. 7:**
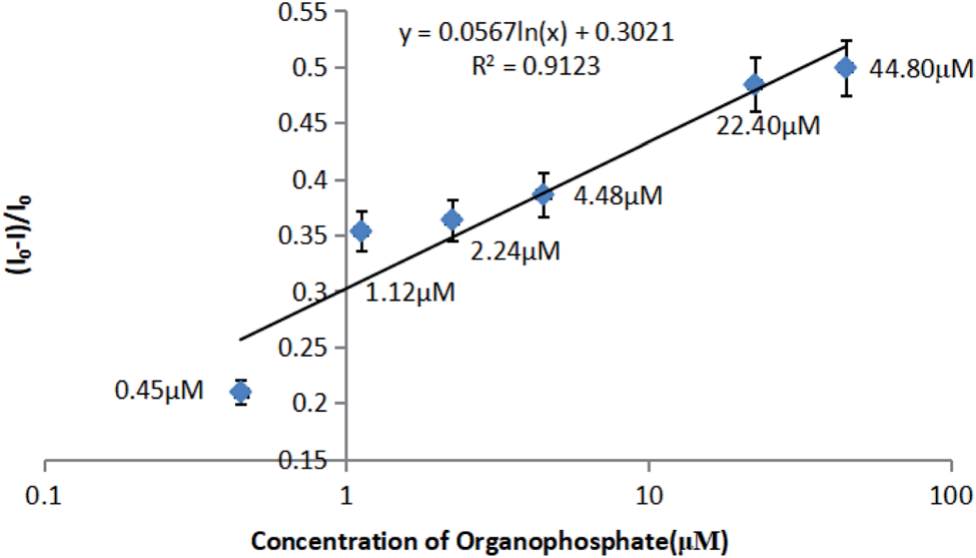
Calibration curve for organophosphate pesticide determination.

The relationship between fluorescence recovery and organophosphate pesticide concentration (Fig. 6d) was used to construct a calibration curve (Fig. 7).

### 3.9 Organophosphate pesticide sensing in real samples

An apple was homogenized and spiked with an organophosphate pesticide (4.48 μM) to afford a positive sample. The equal volume of methanol was added into the spiked sample and stirred violently for sufficiently extraction. Then the samples with extraction solution were centrifuged for 5 min, and the precipitation was discarded. After nitrogen blowing with Termovap Sample Concentrator about 10 min, the methanol was nearly removed. And equal volume of 10% ethanol–PBS complex solution was added into tubes as redissolution liquid. Subsequently, AChE and ATCh were added, and the mixture was incubated for 8 min and treated with CD/Au(iii), followed by fluorescence recovery measurements.

Then according to the correction curve established in 3.8, the concentration of organophosphate can be obtained by calculation.

The obtained data were used to determine the pesticide concentration and recovery as 4.47 μM and 99.85%, respectively, using the abovementioned calibration curve.

The obtained recovery met the requirements of organophosphate pesticide determination, lying in the stipulated range of 80–120%.

## 4 Conclusions

Herein, CDs prepared from egg white by a hydrothermal method were used to fabricate a biosensor for detecting organophosphate pesticides. Initially, the fluorescence of CDs was quenched by a certain amount of Au(iii) ions, which were subsequently scavenged by TCh generated by AChE-catalyzed hydrolysis of ATCh, which resulted in CD fluorescence recovery. The activity of AChE was reduced in the presence of organophosphates, which decreased fluorescence recovery and allowed the pesticide concentration to be determined using a pre-established calibration curve. Tests performed for a real pesticide-spiked sample (4.48 μM) indicated a recovery of 99.85%, revealing that the developed method is well suited for practical applications, with its linear response range corresponding to 0.45–44.80 μM.

## Conflicts of interest

There are no conflicts to declare.

## Abbreviation

AChE: Acetylcholinesterase
ATCh: Acetylthiocholine
CD: Carbon dot
ACh: Acetylcholine
TCh: Thiocholine
DI: Deionized
TEM: Transmission electron microscopy
PBS: Phosphate-buffered saline

## Supplementary Material

RA-008-C7RA13404E-s001
