# Supplementary figures and images for: “Off–On” fluorescent sensing of organophosphate pesticides using a carbon dot–Au(iii) complex
Source: RSC Adv. 2018 Mar 23;8(21):11551–6. doi: 10.1039/c7ra13404e (PMC9079255; doi:10.1039/c7ra13404e)

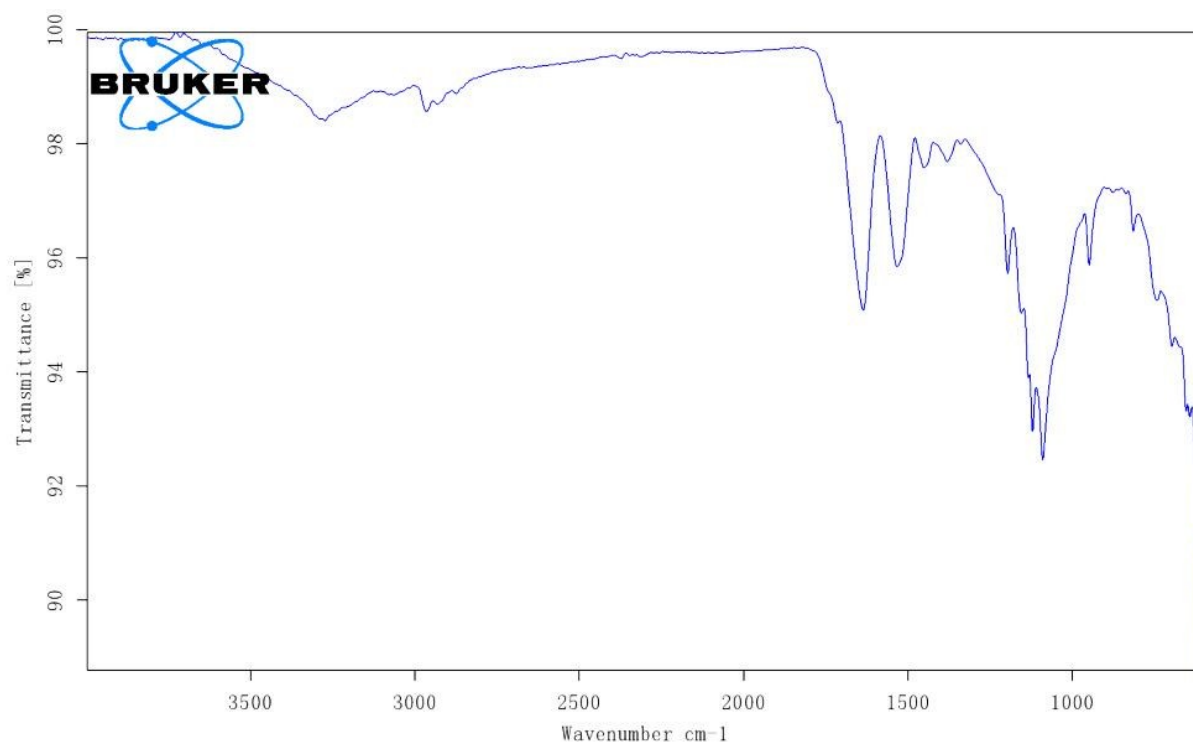

Figure S1

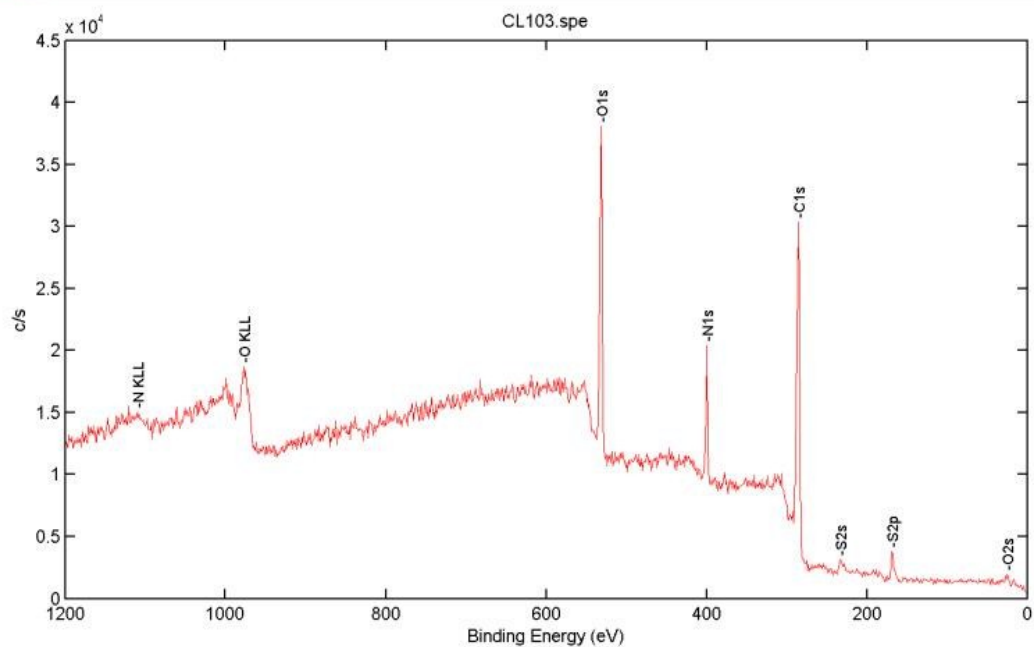

Figure S2

Supplement: RA-008-C7RA13404E-s001 [file RA-008-C7RA13404E-s001.pdf]
